# Rapid eye movement sleep without atonia in patients with sleep-related fronto-temporal epilepsy

**DOI:** 10.1007/s10072-025-08236-1

**Published:** 2025-05-16

**Authors:** Gulcin Benbir Senel, Rumeysa Unkun, Derya Karadeniz, Carlos H. Schenck

**Affiliations:** 1 Sleep and Disorders Unit, Division of Clinical Neurophysiology, Department of Neurology, Cerrahpasa Faculty of Medicine, Istanbul University-Cerrahpasa, Istanbul, Turkey; 2 Minnesota Regional Sleep Disorders Center, Minneapolis, MN USA; 3 Department of Psychiatry, University of Minnesota Medical School, Minneapolis, MN USA

**Keywords:** REM sleep without atonia, Treatment-resistant epilepsy, Interictal epileptiform discharges, Sleep-related hypermotor epilepsy

## Abstract

**Background:**

Interictal epileptiform discharges (IEDs) increase during non-rapid eye movement (NREM) sleep, and decrease or disappear in REM sleep, especially during phasic REM sleep. REM sleep without atonia (RSWA), and its possible effects on IEDs, has not yet been studied.

**Methods:**

A retrospective review of 10-year data retrieved 205 adults with fronto-temporal epilepsy, with full clinical data, 18-channel EEG and polysomnography. Tonic and phasic REM sleep periods were analyzed, and REM atonia was scored with the latest criteria. EEG recordings and IEDs were also re-evaluated in NREM sleep from the first and second halves of the night, and during phasic/tonic REM, and RSWA periods.

**Results:**

RSWA was detected in 31 patients (15.1%) with epilepsy. Total number of IEDs was 18.2 *±* 9.5, being significantly higher in patients with treatment-resistant epilepsy (TRE) than in those without TRE (*p* = 0.046). The number of IEDs was significantly higher in tonic REM than in phasic REM (*p* = 0.001). Patients with RSWA had more IEDs in phasic (*p* = 0.003) and tonic (*p* = 0.037) REM. The number of IEDs in phasic REM periods in patients without RSWA was significantly lower in patients without TRE in compared to those with TRE (*p* = 0.044). IEDs in REM sleep were significantly more common in patients with RSWA, mainly in tonic REM periods, and in patients with TRE.

**Discussion:**

Our data demonstrate for the first time that the suppressing role of REM atonia on IEDs was diminished or lost in the presence of RSWA, including being more prominent in patients with TRE.

## Introduction

Complex bidirectional relationship between sleep and epileptic activities has been observed for many years [1]. It is well-known that the occurrence rate and extent of interictal epileptiform discharges (IEDs) increase in non-rapid eye movement (REM) sleep. In opposite to non-REM sleep, REM sleep decreases the likelihood of occurrence of IEDs, which appear in more focal brain areas in this state [2]. IEDs in REM sleep are therefore valued as an important indicator for the localization of the epileptogenic zone. It is thought to result from thalamocortical desynchronization in REM sleep linked to widespread cholinergic activity [3]. In addition, the inhibitory effects of REM sleep on IEDs were shown to be more prominent in neocortical regions compared to mesiotemporal areas, probably because neuronal desynchronization is majorly driven by neocortical cholinergic network [4]. However, Ho et al. [5] showed that IEDs in neocortical regions were suppressed, and those in mesiotemporal regions were increased in REM sleep compared to wakefulness. When comparing to NREM sleep, IEDs in both areas were decreased in REM sleep, with a more pronounced suppression in mesiotemporal areas.

Recent studies have highlighted that suppressor effect of REM sleep on IEDs was evident in phasic periods of REM sleep relative to tonic REM periods [3, 6–8]. REM sleep alternates between phasic and tonic periods; with bursts of eye movements, hippocampal theta activity, myoclonic contractions in skeletal muscles, and cardiopulmonary irregularities in phasic periods, versus a more quiescent state in between phasic activities as tonic periods [9]. It has been hypothesized that the depolarization in thalamocortical neurons blocking thalamocortical oscillations and the production of acetylcholine were largely raised in phasic REM sleep periods [3, 7, 8]. This difference in REM microstates may explain the strongest suppressing effects of phasic REM sleep periods on IEDs occurrence with respect to tonic REM sleep periods. Reduced connectivity in thalamocortical circuits and lowest firing rates of monoaminergic neurons in REM sleep contribute to decreased likelihood of the spatial and temporal summation of epileptic activities [10].

In addition to asynchronous neuronal activity in REM sleep, which prevents the occurrence and propagation of IEDs, absolute muscle atonia in skeletal muscles in REM sleep contributes preventing the emergence of clinical motor accompaniment. Lesions in pontine areas responsible from REM atonia (primarily the sublaterodorsal tegmental nucleus) in animal models of epilepsy resulted in REM sleep without atonia (RSWA) and more common motor accompaniment in REM sleep when there was an epileptic activity; the low frequency of epileptic activities in REM sleep, however, did not change [3]. RSWA is the pathognomonic electrophysiological biomarker of REM sleep behavior disorder (RBD) [11]. Data regarding RBD in patients with epilepsy is very scarce. Manni et al. [12] reported that undiagnosed or misdiagnosed isolated RBD coexisted in 12.5% of eighty patients with epilepsy. The authors raised the question whether, or not, this was an incidental comorbidity due to increased prevalence of both RBD and epilepsy with ageing, and proposed further studies to establish the extent of pathophysiological interactions between epilepsy and RBD. On the other hand, it was demonstrated that cortical desynchronization in REM sleep was also impaired in patients with RBD [13] in addition to RSWA. It may therefore be speculated that, contrary to the protective role of REM sleep on epileptic activities– mainly during phasic REM periods, RBD might indeed facilitate the occurrence of IEDs and sleep-related seizures. In this study, we evaluated the features of REM sleep and the presence of RSWA in patients with sleep-related hypermotor epilepsy, and investigated the relationship between the characteristics of RSWA, IEDs and epilepsy.

## Methods

### Patient selection

We retrospectively scanned our last 10-year data for the patients analyzed with 18-channel electroencephalography (EEG) and polysomnography (PSG) in our sleep laboratory with a pre-diagnosis of sleep-related epilepsy and/or the complaints of abnormal motor movements during sleep. A total of 739 patients were detected; of these, patients lacking definitive diagnosis and those with incomplete data were excluded. A total of 327 patients had a definitive diagnosis of epilepsy with full clinical and electrophysiological assessments. Of these, patients below the age of 18 years (*n* = 122) were also excluded. EEG and PSG characteristics of 205 adult patients with sleep-related hypermotor (SHE) epilepsy were re-analyzed. The study was approved by the Local Ethics Committee in Istanbul University-Cerrahpasa (E-83045809-604.01-992867), and performed in accordance with the Declaration of Helsinki and its later amendments. As it was conducted in a retrospective design, informed consent was waived by the Local Ethics Committee.

### Clinical and PSG examinations

Clinical and PSG data of 205 adult patients with epilepsy were analyzed. Clinical data included the demographics of the patients, sex, age on admission, and body mass index (BMI). The use of anti-seizure medications was interrogated in detail. The diagnosis of SHE was confirmed based on its diagnostic criteria as defined by Tinuper et al. [14]. Treatment-resistant epilepsy (TRE) was defined on the basis of the consensus made by the International League Against Epilepsy (ILAE) as failure of seizure freedom in spite of trials of two anti-seizure medications chosen appropriately for the seizure type, tolerated and titrated up to optimal maximal doses, and tried alone or in combination with other anti-seizure medications [15].

A full-night PSG recording had been performed in all patients in sleep laboratory with chin electromyography (EMG) and bilateral anterior tibialis EMG recordings. In PSG recordings of past two years, additional bilateral flexor digitorium superficialis EMG was also used, as the additive value of the recordings of muscular activity in this muscle were defined [16]. Sleep and related parameters were re-scored visually by the European Sleep Expert (G.B.S.), on the basis of the latest version of the American Academy of Sleep Medicine (AASM) Manual for the Scoring of Sleep and Associated Events [17]. PSG parameters included total recording time (TRT), total sleep time (TST), sleep latency (SL), REM sleep latency (REML), sleep efficiency (SE), wakefulness after sleep onset (WASO), duration and percentage of sleep stages, apnea-hypopnea index (AHI), minimum and mean oxygen saturations, and hourly index of periodic leg movements in sleep (PLMSI).

REM atonia was also scored according to the latest criteria as follows: (1) excessive sustained (tonic) muscle activity in chin EMG with an amplitude of at least 2 times greater than REM atonia lasting for at least 50% of the duration of the epoch, or (2) excessive transient (phasic) muscle activity each lasting more than five seconds and being in at least half of the 10 sequential 3-seconds mini-epochs in chin and/or extremity EMG, or (3) any chin EMG activity with an amplitude of at least 2 times greater than REM atonia including bursts lasting 5 to 15 s in chin and/or extremity EMG [15]. REM sleep period was then divided into two as phasic and tonic REM sleep; phasic REM sleep was scored in the presence of bursts of rapid eye movements clearly distinguishable on the electrooculography channels lasting for at least 3 s [7, 18], while the periods in between were regarded as tonic REM sleep.

### EEG evaluations

All patients had 18-channel scalp EEG with standardized bipolar montages in accordance with international 10–20 system [19]. Of 205 adult patients with SHE, 125 patients had normal wakefulness and sleep EEG, and eight patients had generalized slowing in background activity interfering with sleep scoring in PSG recordings. Four patients had epileptic seizures during their PSG recordings. Four patients did not have at least five minutes of unfragmanted REM sleep duration in the first and second halves of the night. At the end, EEG features and the characteristics of interictal discharges were reevaluated in 64 adult patients with SHE. They all had a well-defined epileptic zone, and were seizure-free for the last 24 h. Five minutes of unfragmanted REM and NREM (N2) sleep durations from the first and second halves of the night were selected considering the circadian modulation of sleep stages [20], and visually analyzed for the interictal epileptiform discharges (IEDs). The number of IEDs was calculated as IEDs occurring independently in all EEG channels, and separately for NREM and REM sleep periods, for phasic and tonic REM sleep, and also for periods with RSWA.

### Statistical analysis

Data was analyzed by using SPSS (Statistical Package for the Social Sciences) version 21.0 (SPSS Inc., Chicago, IL, USA). Nominal parameters were given in numbers and percentages, and were compared by using chi-square test. Numeric parameters were first tested by Shapiro-Wilk test to be determined if they were normally-distributed or not. Accordingly, independent-t test or Mann-Whitney U test was used in comparative analyses. Continuous parameters were given in mean *±* standard deviation or as median [interquartile range] for the normally and non-normally distributed values, respectively. Dependent parameters were tested by using paired sample t test or Wilcoxon test, in case of non-normality. One-was ANOVA (Analysis of Variance) was performed for the multiple comparisons with post-hoc LSD analysis. Multiple comparisons were also corrected by Benjamin-Hochberg procedure, and a value of equal to or below 0.05 was accepted as statistically significant.

## Results

Out of 205 patients with SHE (with a mean age of 42.0 *±* 15.6 years; and 108 males, 52.7%), 31 patients (15.1%) had RSWA, while none of them had an RBD attack during PSG recordings or a typical history of RBD. In comparison to patients with and without RSWA, 64.5% were men in epileptic patients with RSWA, and 50.6% were men in those without (*p* = 0.108). The mean age of the patients with RSWA was 47.4 *±* 16.8 years, while it was 41.0 *±* 15.3 years in patients without RSWA (*p* = 0.061). BMI was similar between two groups of patients. RSWA was more common in patients with TRE than in those without, though not significantly (9.1% versus 5.6% accordingly, *p* = 0.522).

PSG parameters (Table 1) showed that REM sleep duration was significantly longer in patients with RSWA (median, 16.0 [13.0-18.5]; mean *±* s.d., 15.7 *±* 7.6; %) compared to those without RSWA (median, 13.0 [11.8–14.5]; mean *±* s.d., 13.2 *±* 8.6; %) (*p* = 0.045). Total PLMSI was non-significantly higher in patients with RSWA (21.4 *±* 25.3/hour versus 12.8 *±* 19.8/hour, respectively; *p* = 0.244). In contrast, PLMSI in REM sleep was significantly higher in patients with RSWA (16.0 *±* 11.3/hour) than those without RSWA (7.1 *±* 7.8/hour, *p* = 0.042).

**Table 1:** PSG parameters between patients with and without RSWA in whole study population (*n* = 205)

PSG parameters	Patients without RSWA	Patients with RSWA	*p* value
TRT (min)	369.5 *±* 77.4	387.2 *±* 55.9	0.490
TST (min)	25.7 *±* 45.3	21.4 *±* 29.1	0.225
SL (min)	164.7 *±* 91.6	143.8 *±* 95.3	0.200
REML (min)	76.1 *±* 14.5	79.8 *±* 9.4	0.298
SE (%)	80.6 *±* 54.9	89.4 *±* 49.8	0.203
WASO (min)	8.9 *±* 6.3	10.1 *±* 5.8	0.265
N1 sleep (%)	44.0 *±* 13.0	44.0 *±* 10.5	0.335
N2 sleep (%)	14.3 *±* 9.4	12.8 *±* 7.7	0.897
N3 sleep (%)	369.5 *±* 77.4	387.2 *±* 55.9	0.386
REM sleep (%)	13.0 [11.8–14.5]	16.0 [13.0-18.5]	0.045*
AHI	23.3 *±* 20.6	22.2 *±* 20.0	0.820
Arousal index	6.1 *±* 3.9	6.0 *±* 4.1	0.896
Mean oxygen saturation	95.1 *±* 1.5	94.4 *±* 1.9	0.091
Minimum oxygen saturation	83.6 *±* 7.8	83.5 *±* 6.2	0.928
PLMSI	12.8 *±* 19.8	21.4 *±* 25.3	0.244
PLMSI– REM sleep	7.1 *±* 7.8	16.0 *±* 11.3	0.042*

PSG: polysomnography; RSWA: REM (rapid eye movement) sleep without atonia; TRT: total recording time; TST: total sleep time; SL: sleep latency; REML: REM sleep latency; WASO: wakefulness after sleep onset; AHI: apnea-hypopnea index; PLMSI: Index of periodic leg movements in sleep

Data given in mean *±* standard deviation for normally distributed parameters, in median [interquartile range] for non-normally distributed parameters

*statistically significant

EEG was normal in 75.0% of patients with RSWA, and in 54.7% of patients without RSWA (*p* = 0.075). To evaluate the presence of IEDs in detail in corresponding to the sleep stage from which they arise, and also in comparing phasic and tonic REM sleep, patients with normal wakefulness and sleep EEG, those with generalized slowing in background activity interfering sleep scoring, those lacking at least five minutes of unfragmanted REM sleep in the first and second halves of the night, and patients having epileptic seizures at PSG night were excluded. In remaining 64 adult patients with SHE, six patients (9.4%) had RSWA. In this subgroup of epileptic patients with localized IEDs in PSG plus EEG recordings, men to women ratio (4 men, 66.7% versus 31 men, 53.4% respectively; *p* = 0.431), and the mean age (48.0 *±* 5.3 years versus 39.2 *±* 1.8 years respectively; *p* = 0.157) were higher in patient with RSWA, but not statistically different between patients with and without RSWA. PSG data (Table 2) showed that sleep latency was much shorter in patients with RSWA (5.9 *±* 5.2 min) than those without (14.5 *±* 15.6 min, *p* = 0.010). REM sleep duration was longer in patients with RSWA, but the difference was not significant (Table 2). PLMSI and PLMSI in REM sleep were higher in patients with RSWA (10.5 *±* 10.1/hour and 10.5 *±* 5.0/hour, respectively) than in those without RSWA (9.0 *±* 16.2/hour and 5.2 *±* 0.6/hour, respectively) (*p* = 0.812 and *p* = 0.043, respectively).

**Table 2:** Comparison of PSG parameters in patients with epilepsy and interictal discharges in EEG recordings (*n* = 64) with and without RSWA

PSG parameters	Patients without RSWA	Patients with RSWA	*p* value
TRT (min)	487.6 *±* 40.2	490.1 *±* 25.9	0.882
TST (min)	382.3 *±* 58.0	389.0 *±* 72.2	0.793
SL (min)	14.5 *±* 15.6	5.9 *±* 5.2	0.010*
REML (min)	158.9 *±* 89.0	145.6 *±* 74.0	0.696
SE (%)	79.1 *±* 10.9	83.8 *±* 10.1	0.326
WASO (min)	92.0 *±* 60.7	79.1 *±* 50.2	0.577
N1 sleep (%)	7.7 *±* 5.3	9.1 *±* 5.6	0.564
N2 sleep (%)	44.7 *±* 12.0	48.7 *±* 11.0	0.437
N3 sleep (%)	15.4 *±* 10.5	12.1 *±* 5.7	0.254
REM sleep (%)	13.0 [11.8–15.2]	14.0 [7.0-21.6]	0.761
AHI	14.6 *±* 9.6	9.2 *±* 5.6	0.134
Arousal index	7.2 *±* 5.3	3.0	0.465
Mean oxygen saturation	95.3 *±* 1.5	94.9 *±* 2.9	0.615
Minimum oxygen saturation	84.4 *±* 6.4	88.0 *±* 4.2	0.299
PLMSI	9.0 *±* 16.2	10.5 *±* 10.1	0.812
PLMSI– REM sleep	5.2 *±* 0.6	10.5 *±* 5.0	0.043*

PSG: polysomnography; RSWA: REM (rapid eye movement) sleep without atonia; TRT: total recording time; TST: total sleep time; SL: sleep latency; REML: REM sleep latency; WASO: wakefulness after sleep onset; AHI: apnea-hypopnea index; PLMSI: Index of periodic leg movements in sleep

Data given in mean *±* standard deviation for normally distributed parameters, in median [interquartile range] for non-normally distributed parameters

*statistically significant

**Fig. 1:**
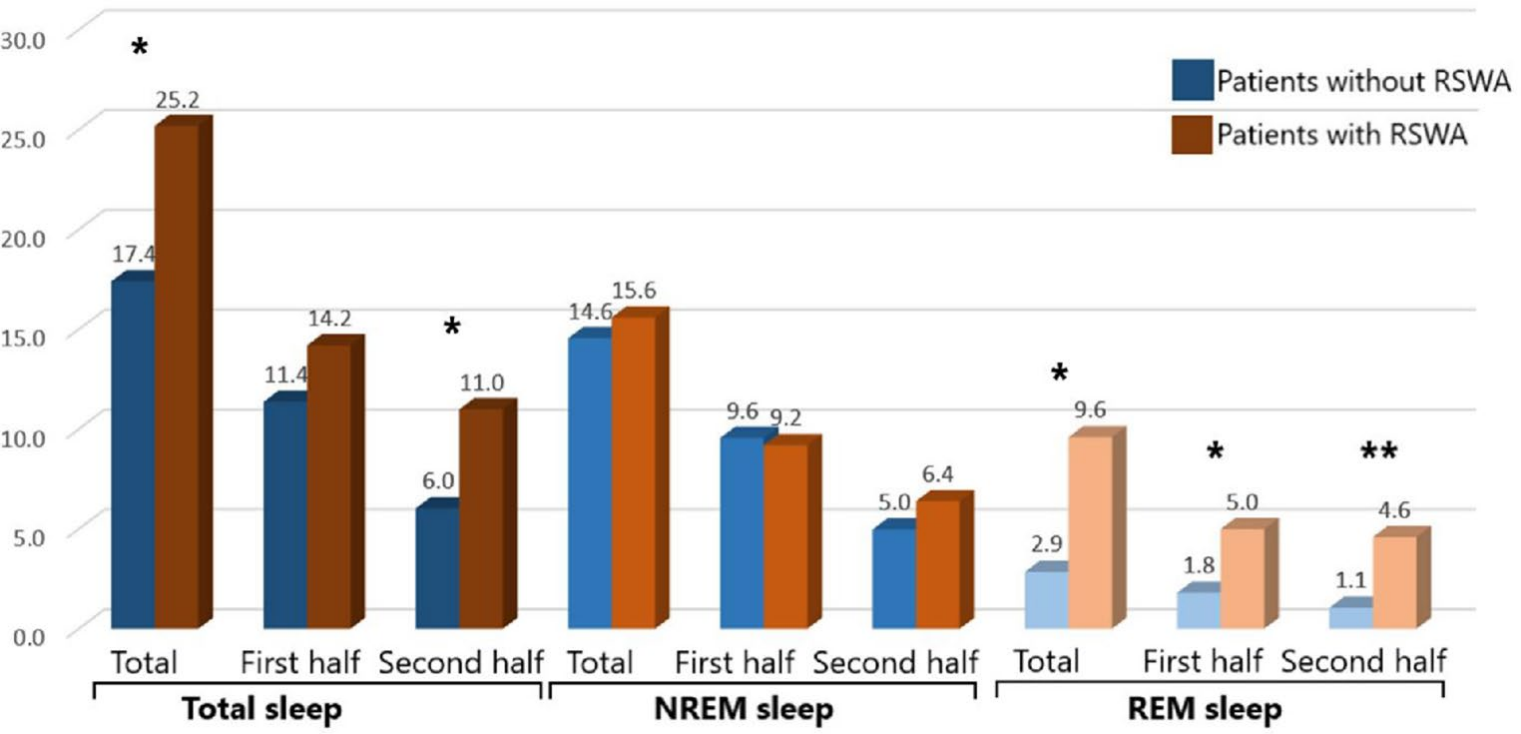
Numbers of IEDs in different sleep periods in patients with and without RSWA (*p *≤* 0.05, **p *≤* 0.01)

Total number of IEDs was 18.2 *±* 9.5 (range, 2–42) in the whole population, being significantly higher in patients with TRE than those without (25.6 *±* 15.0 and 17.2 *±* 8.4, respectively; *p* = 0.046). Two out of six patients with RSWA (33.3%) versus four patients out of 58 patients (6.9%) without RSWA had TRE (*p* = 0.035). Total number of IEDs was also higher in patients with RSWA in compared to those without (25.2 *±* 9.7 and 17.4 *±* 9.2, respectively), but the difference failed to reach to the statistically significant level (*p* = 0.077). IEDs analysis in NREM and REM sleep periods in the first and the second halves of the night and total numbers are shown in Fig. 1. Here it was observed that the number of IEDs in total sleep periods (*p* = 0.047), in the second half of sleep periods (*p* = 0.025), in total REM sleep period (*p* = 0.004), and in REM sleep in the first (*p* = 0.024) and second (*p* = 0.001) halves of the night were significantly higher in patients with RSWA.

**Fig. 2:**
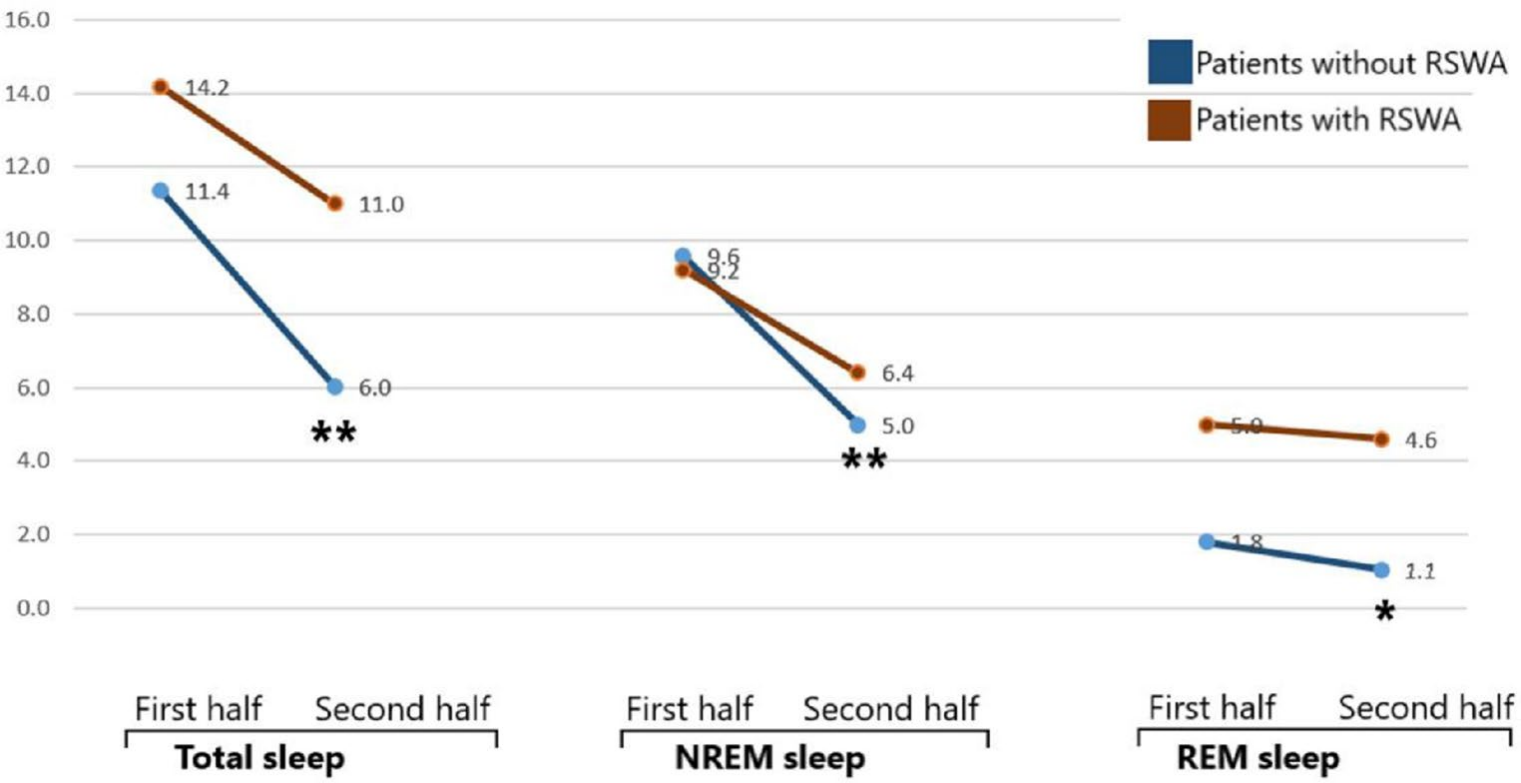
Change in IEDs in the second half of the night in compared to the first half in patients with and without RSWA (*p = 0.001, **p < 0.001)

**Fig. 3:**
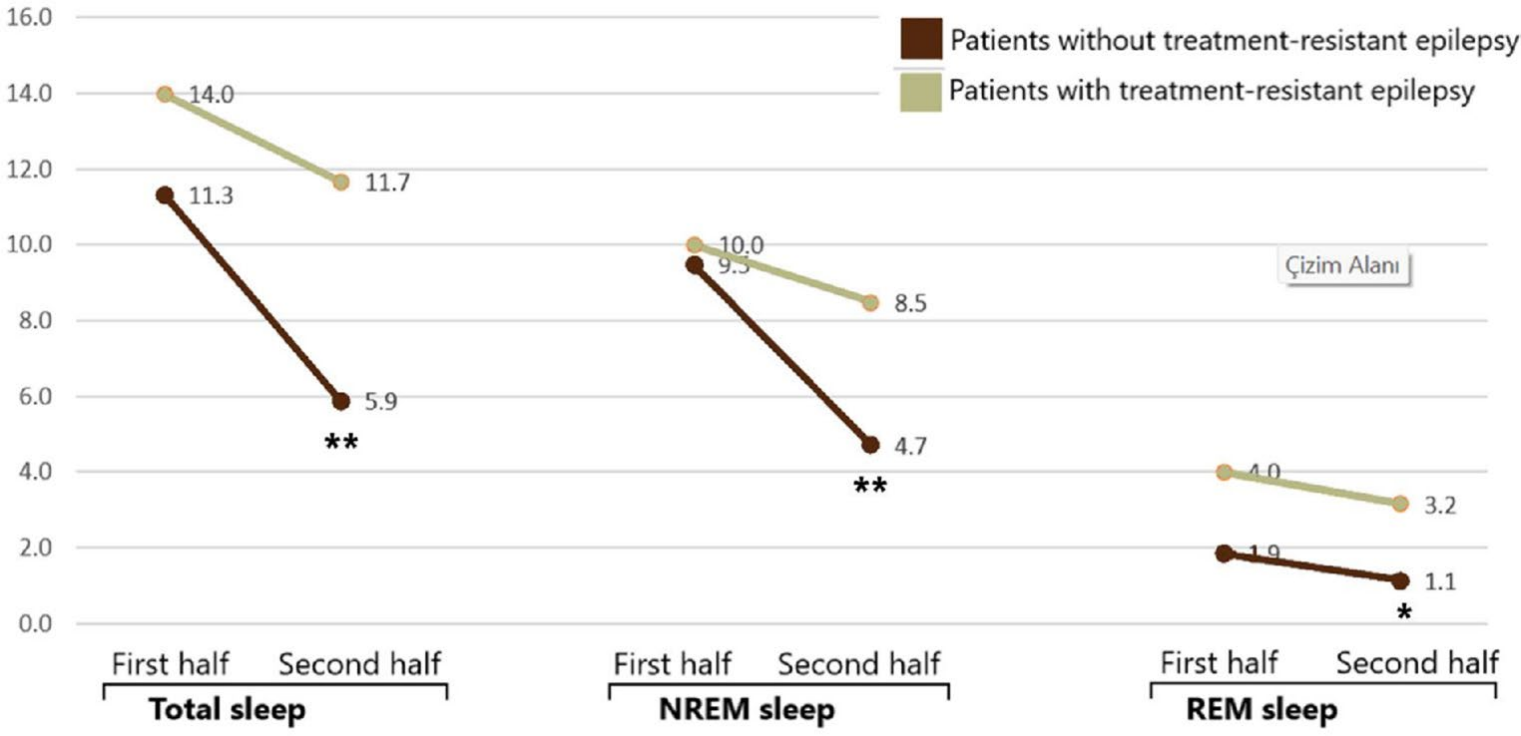
Change in IEDs in the second half of the night in compared to the first half in patients with and without TRE

The numbers of IEDs in the second half of the night was decreased in compared to the first half of the night (Fig. 2), which was highly significant in patients without RSWA in all sleep periods (*p* < 0.001 for total and NREM sleep periods, *p* = 0.001 for REM sleep periods). On the other hand, the decrease in the numbers of IEDs in patients with RSWA was not significant (*p* = 0.253 for total sleep periods, *p* = 0.087 for NREM sleep periods and *p* = 0.778 for REM sleep periods). The change in the numbers of IEDs was also analyzed between patients with and without TRE (Fig. 3). A similar pattern was here observed so that in patients without TRE, the decrease in IEDs in the second half of the night was highly significant (*p* < 0.001 for total and NREM sleep periods, *p* = 0.001 for REM sleep periods), while this was not the case in patients with TRE (*p* = 0.302 for total sleep periods, *p* = 0.151 for NREM sleep periods and *p* = 0.571 for REM sleep periods).

**Fig. 4:**
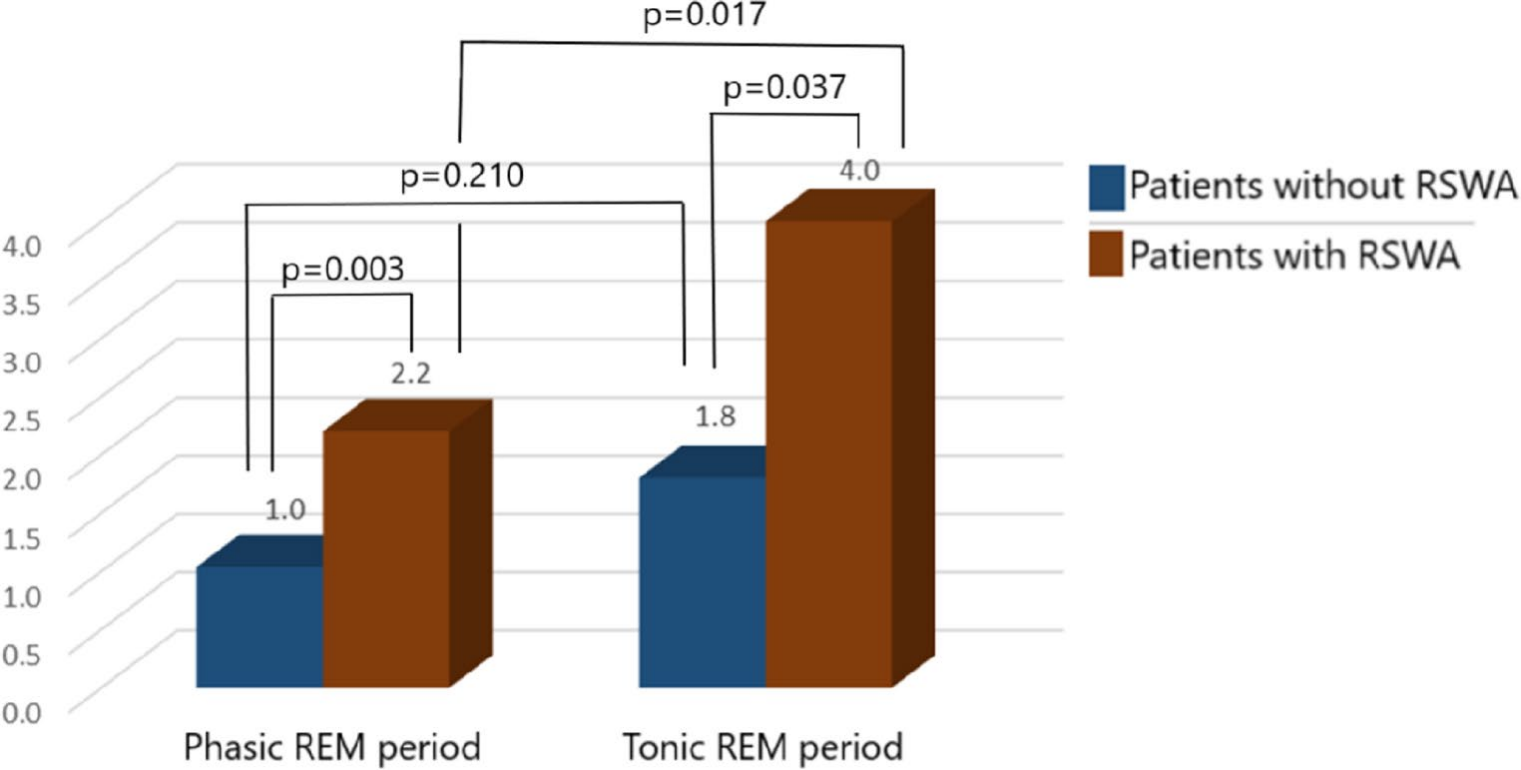
Distribution of IEDs in phasic and tonic REM sleep periods in patients with and without RSWA

**Fig. 5:**
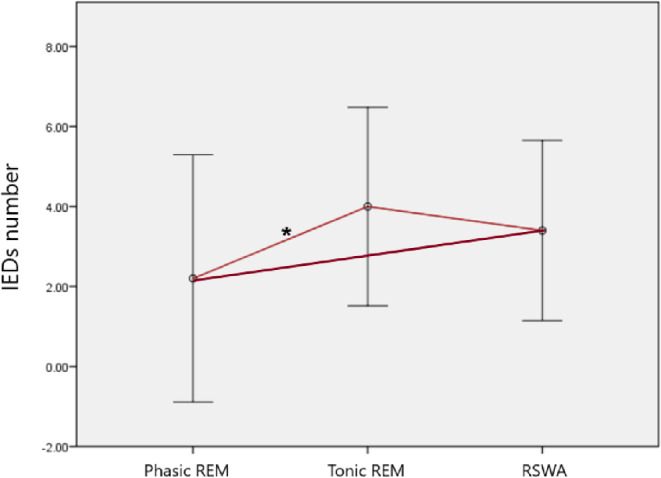
Distribution of interictal discharges in phasic, tonic and loss of atonia periods in six patients with RSWA

REM sleep period was also divided into two as phasic and tonic REM periods, and it was observed that the number of IEDs was significantly higher in tonic REM periods than those in phasic REM periods (2.0 *±* 3.0 versus 1.1 *±* 1.9, *p* = 0.001). The distribution of IEDs among phasic and tonic REM periods was also analyzed separately in patients with and without RSWA (Fig. 4), and it was demonstrated that patients with RSWA had more IEDs both in phasic (*p* = 0.003) and tonic (*p* = 0.037) REM periods in compared to those without RSWA. On the other side, the difference in the number of IEDs occurring in phasic and tonic REM sleep periods was statistically significant only in patients without RSWA (*p* = 0.017). Although IEDs were also occurring more commonly in tonic REM sleep periods in patients without RSWA, the difference failed to reach to the statistically significant level (*p* = 0.210, Fig. 4). Only in patients with RSWA (*n* = 6), we analyzed the distribution of IEDs in between phasic REM sleep, tonic REM sleep and REM sleep without atonia periods, separately (Fig. 5). The highest number of IEDs was present in tonic REM sleep period, and the lowest number of IEDs was present in phasic REM sleep period; IEDs occurring in RSWA periods, on the other hand, were placed in between. The only significant difference was observed between the numbers of IEDs occurring in phasic versus tonic REM sleep periods (*p* = 0.037), while those between phasic REM sleep versus RSWA (*p* = 0.261) or tonic REM sleep versus RSWA (*p* = 0.374) were not statistically significant.

**Fig. 6:**
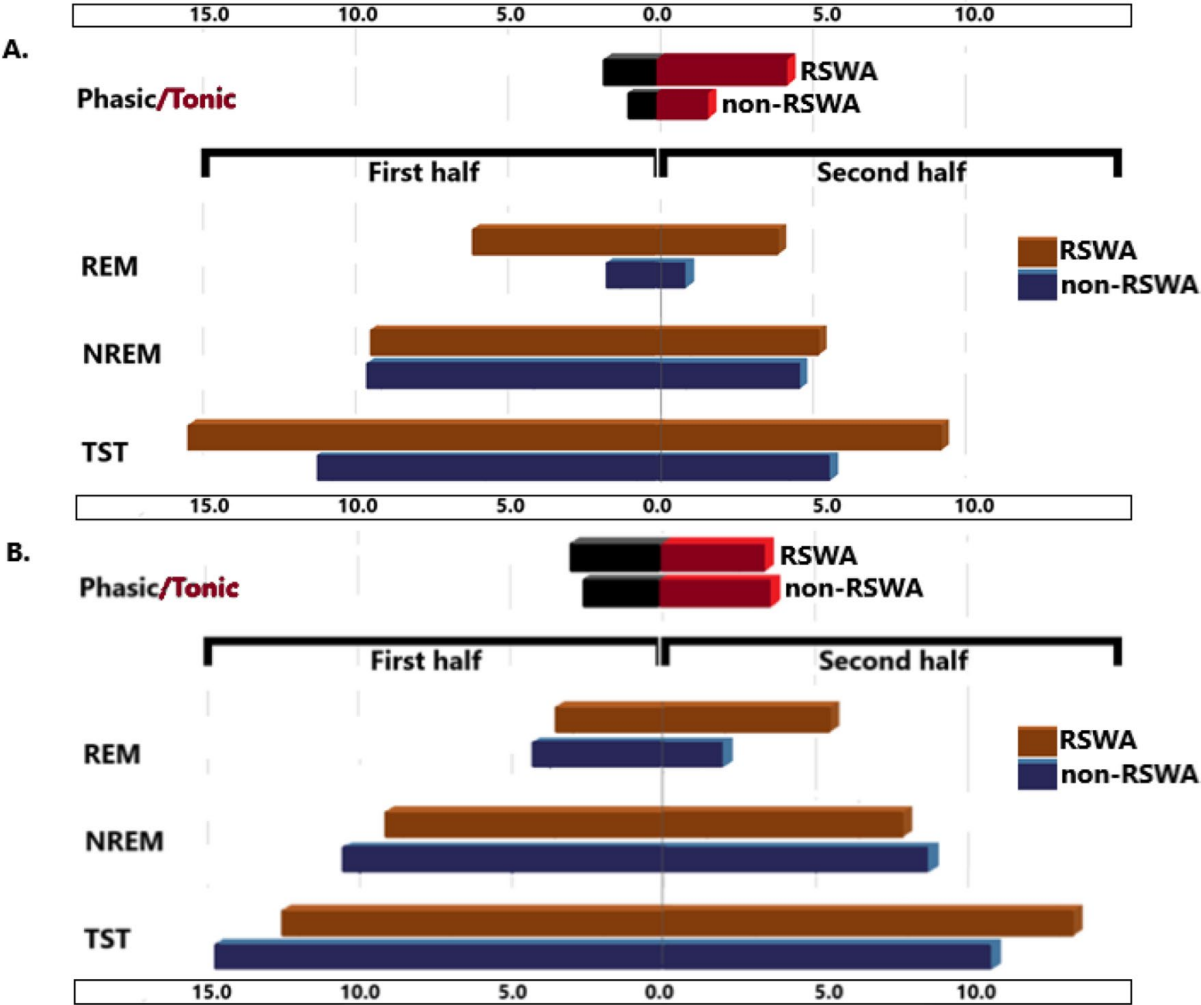
Numbers of IEDs in patients without (**A**) and with TRE (**B**) according to the presence of RSWA, in the first and second halves of the sleep, in different sleep stages, and in phasic and tonic REM periods. In patients without TRE, the appearance of IEDs was mainly detected in the first half of the night, both in NREM and REM sleep (*p* < 0.05), while this difference in the numbers of IEDs between the first and second halves of the night was lost in patients with TRE. In patients without TRE, IEDs in REM sleep were significantly more common in patients with RSWA, and mainly observed in tonic REM periods. These differences were lost in patients with TRE

We then compared the changes in IEDs between first and second halves of the night in different sleep stages and in between patients with and without RSWA (see Fig. 6) in two groups of patients with and without TRE. Here, the decrease in IEDs in the second half of the night was clearly seen in patients without TRE (Fig. 6A), which was not observed in those with TRE (Fig. 6B). In patients without RSWA, the decrease in IEDs in the second half of the night was significantly more pronounced in patients without TRE for the total sleep (*p* = 0.017) and for NREM sleep (*p* = 0.006) periods, in compared to those with TRE, while it was not significant for REM sleep period (*p* = 0.407). None of these was not statistically significantly different between those with and without TRE in patients with RSWA (*p* = 0.341 for total sleep, *p* = 0.386 for NREM and *p* = 0.265 for REM sleep periods). The number of IEDs in phasic REM periods in patients without RSWA was significantly lower (*p* = 0.044) in patients without TRE in compared to those with TRE. The number of IEDs in tonic REM periods was also lower in patients without TRE in compared to those with TRE in patients without RSWA, but not significantly (*p* = 0.063). The differences in the numbers of IEDs in phasic and tonic REM sleep periods between patients with and without TRE, however, were not different in case of RSWA (*p* = 0.632 and *p* = 0.712, respectively).

## Discussion

Although the reciprocal relationship between epilepsy and REM sleep has been extensively studied, to our knowledge this is the first study to investigate the presence and associated effects of RSWA in patients with sleep-related hypermotor epilepsy. While REM sleep is known to play a protective role against IEDs and seizures owing to its atonic EMG characteristics and asynchronous EEG activity– mainly during phasic REM, we demonstrated the novel important finding that the loss of atonia in REM sleep was associated with an increased number of IEDs, and treatment-resistance in patients with SHE.

The overall prevalence of RSWA was observed to be circ. 15% in patients with epilepsy, including 9.4% in patients with SHE, having localized IEDs in the same PSG plus EEG recording. The overall PLMSI and PLMSI in REM sleep were higher in patients with RSWA, as previously reported in non-epileptic patients with RSWA and/or RBD [21, 22]. There is only one study in the literature that examined the presence of isolated RBD in epilepsy patients aged over 60 years, reporting a prevalence of 12.5%, more likely to occur in men with cryptogenic epilepsy and sleep-related seizures [12]. No data were reported on PSG parameters or details on phasic-tonic REM sleep periods. Considering the prevalence of epilepsy and RSWA in the general population [23, 24], our data suggest that the coexistence of epilepsy and RSWA indicates causal comorbidity with shared pathophysiological interactions.

Despite the modest sample size, we found that the presence of TRE was much more common in patients with RSWA (33.3%) compared to those without RSWA (6.9%). In patients with RSWA, there were significantly higher numbers of total IEDs and IEDs in the second half of sleep, along with total IEDs in REM sleep, and in both REM sleep periods during the first and second halves of the night. While there was a significant decrease of IEDs in the second half of the night in patients with SHE without RSWA– as a known physiologic phenomenon in epilepsy [5], it was not observed in patients with RSWA. This loss of the physiologic decrease in IEDs in the second half of the night was also found in patients with TRE. However, in patients without RSWA, the decrease in IEDs in total sleep and in NREM sleep periods in the second half of the night was significantly more pronounced in patients without TRE in compared to those with TRE. In patients with RSWA, on the other hand, the difference between patients with and without TRE was no longer significant. These findings show a significant association between RSWA and increased IEDs, disturbed distribution of IEDs throughout the night, and the presence TRE. We may hypothesize that the impaired physiological components of REM sleep in RSWA and/or RBD (namely, the desynchronization of neuronal discharges and muscular atonia [13]) may reflect an ongoing neurodegenerative process, which, in turn, might facilitate the increased occurrence of IEDs, and resistance to treatment.

Our analysis of the occurrence of IEDs in regards to phasic and tonic periods of REM sleep supports previous reports [6–9] demonstrating that IEDs were significantly higher in tonic REM sleep periods. On the other hand, our analysis showed that the patients with RSWA had more IEDs not only in the tonic phase of REM sleep, but also in the phasic phase of REM sleep, so that the difference between tonic and phasic REM sleep periods was lost in these patients with RSWA. In patients with and without TRE, the difference in the numbers of IEDs in phasic versus tonic REM sleep periods was significant in patients without RSWA; the number of IEDs in phasic REM periods was significantly higher in TRE, but the number of IEDs in tonic REM periods was not. In patients with RSWA, however, this difference between patients with and without TRE was also lost, and the numbers of IEDs in phasic and tonic REM sleep periods were similar. While our data support the suppressing role of REM atonia on IEDs, especially during phasic REM periods, it uniquely shows that this effect was diminished or lost in the presence of RSWA, being more prominent in patients with TRE.

In patients with epilepsy, no reduction was found in the overall duration of phasic REM sleep, but phasic REM sleep in the first half of the night was decreased [25]. In patients with refractory epilepsy, however, the duration of total REM sleep during the first and second halves of the night, and particularly the duration of phasic REM sleep, were significantly decreased [26]. As discussed above, increased production of acetylcholine and the depolarization in thalamocortical neurons diminishing neuronal connectivity particularly during phasic REM sleep periods were proposed to be responsible from the strongest suppressing effects of REM sleep on IEDs occurrence, with the additional contribution of absolute muscle atonia, preventing the emergence of clinical motor manifestations [3, 7, 8, 10]. One may assume that the opposite conditions would facilitate the occurrence of IEDs and seizures by reversing these protective properties. Yet, it appears that more complex mechanisms play a role in the pathophysiologic interaction between RSWA and epilepsy, changing the whole dynamics of sleep and treatment-resistant epilepsy.

There are some studies investigating the dynamic change of cortical and thalamic neuronal networks by giving electrical stimulation to the human cortex during REM sleep [27, 28]. These studies showed that both phasic and tonic REM sleep periods displayed a strong suppression on neuronal synchrony, having electric activity patterns and functional connectivity similar to that of wakefulness. However, a unique heterogeneity of phasic and tonic REM sleep periods was observed in cortical and thalamic activity and thalamocortical networks. Higher alpha (α) and beta (β) frequency powers in tonic REM sleep, as an intermediate state in between phasic REM sleep and wakefulness, was related to the regulation of arousal and vigilance states, modulating alertness to environmental stimuli. This tendency in shifting to wakefulness activity in thalamocortical networks– being more easy in tonic REM compared to phasic REM– may explain, in part, the different electrophysiological properties of tonic REM sleep on the occurrence of IEDs.

Furthermore, changes in neuropeptide availability, mainly involving hypocretin, may also play a crucial pathophysiologic link between RSWA and epilepsy. Hypocretin-producing neurons, located in the lateral hypothalamus, have a wide connection with the brainstem structures modulating motor activity, and decreased hypocretinergic activation plays a critical role in the loss of muscle atonia and RBD, especially in patients with narcolepsy [29]. Further support for the human clinical finding of the strong association of narcolepy with RBD (present in up to 60% of narcolepsy-cataplexy patients [30]) has recently been reported in prepro-hypocretin knockout mice, a recognized mouse model of NT type 1, with additional targeted suppression of the sublaterodorsal glutamatergic neurotransmission, a recognized rodent model of iRBD [31]. In this combined experimental mouse narcolepsy-RBD model, there was a significant alteration of tonic and phasic components of the EMG during REM sleep, with more phasic events and more REM sleep episodes without atonia compared to wild-type mice. The importance of these findings was discussed in an accompanying editorial [32]. On the other hand, hypocretinergic neurons also have strong excitatory projections to the cortical brain areas, and increased hypocretinergic activation was suggested to trigger epileptic discharges and seizures in animal models [33]. Thus, impairments in hypocretin-related network pathways may result in RSWA and facilitate epileptic cortical activities, which requires further investigations to reveal possible neurobiological links and pathophysiologic mechanisms.

The identification and further investigation of REM sleep disturbances in patients with epilepsy will open new avenues in the deeper understanding and management of sleep-related seizures and epilepsy. The presence of REM sleep without atonia and/or RBD should be further explored in larger prospective cohorts. Long-term follow-up in these patients is also of crucial importance to delineate any underlying ongoing neurodegenerative process, and the possible prognostic effects of RSWA in epileptic patients.

## References

[1] Rossi GF, Colicchio G, Pola P (1984) Interictal epileptic activity during sleep: a stereo-EEG study in patients with partial epilepsy. Electroencephalogr Clin Neurophysiol 58:97–106. 10.1016/0013-4694(84)90022-1 6204846 10.1016/0013-4694(84)90022-1

[2] Frauscher B, von Ellenrieder N, Dubeau F, Gotman J (2016) EEG desynchronization during phasic REM sleep suppresses interictal epileptic activity in humans. Epilepsia 57(6):879–888. 10.1111/epi.13389 27112123 10.1111/epi.13389 PMC4949560

[3] Shouse MN, Farber PR, Staba RJ (2000) Physiological basis: how NREM sleep components can promote and REM sleep components can suppress seizure discharge propagation. Clin Neurophysiol 111:S9–S18. 10.1016/s1388-2457(00)00397-7 10996550 10.1016/s1388-2457(00)00397-7

[4] Fouad A, Azizollahi H, Le Douget JE, Lejeune FX, Valderrama M, Mayor L, Navarro V, Le Van Quyen M (2022) Interictal epileptiform discharges show distinct Spatiotemporal and morphological patterns across wake and sleep. Brain Commun 4(5):fcac183. 10.1093/braincomms/fcac183 36483575 10.1093/braincomms/fcac183 PMC9724782

[5] Ho A, Hannan S, Thomas J, Avigdor T, Abdallah C, Dubeau F, Gotman J, Frauscher B (2023) Rapid eye movement sleep affects interictal epileptic activity differently in mesiotemporal and neocortical areas. Epilepsia 64(11):3036–3048. 10.1111/epi.17763 37714213 10.1111/epi.17763

[6] Busek P, Buskova J, Nevsimalova S (2010) Interictal epileptiform discharges and phasic phenomena of REM sleep. Epileptic Disord 12(3):217–221. 10.1684/epd.2010.0319 20630820 10.1684/epd.2010.0319

[7] Giacomini T, Luria G, D’Amario V, Croci C, Cataldi M, Piai M, Nobile G, Bruni O, Consales A, Mancardi MM, Nobili L (2022) On the role of REM sleep microstructure in suppressing interictal spikes in electrical status epilepticus during sleep. Clin Neurophysiol 136:62–68. 10.1016/j.clinph.2022.01.008 35139436 10.1016/j.clinph.2022.01.008

[8] Campana C, Zubler F, Gibbs S, de Carli F, Proserpio P, Rubino A, Cossu M, Tassi L, Schindler K, Nobili L (2017) Suppression of interictal spikes during phasic rapid eye movement sleep: a quantitative stereo-electroencephalography study. J Sleep Res 26(5):606–613. 10.1111/jsr.12533 28401614 10.1111/jsr.12533

[9] Simor P, van der Wijk G, Gombos F, Kovács I (2018) The paradox of rapid eye movement sleep in the light of oscillatory activity and cortical synchronization during phasic and tonic microstates. NeuroImage 202:116026. 10.1016/j.neuroimage.2019.116066 10.1016/j.neuroimage.2019.116066 31377324

[10] Ng M, Pavlova M (2013) Why are seizures rare in rapid eye movement sleep? Review of the frequency of seizures in different sleep stages. Epilepsy Res Treat 2013:932790. 10.1155/2013/932790 23853720 10.1155/2013/932790 PMC3703322

[11] Schenck CH, Bundlie SR, Ettinger MG, Mahowald MW (1986) Chronic behavioral disorders of human REM sleep: a new category of parasomnia. Sleep 9(2):293–308. 10.1093/sleep/9.2.293 3505730 10.1093/sleep/9.2.293

[12] Manni R, Terzaghi M, Zambrelli E (2007) REM sleep behaviour disorder in elderly subjects with epilepsy: frequency and clinical aspects of the comorbidity. Epilepsy Res 77(2–3):128–133. 10.1016/j.eplepsyres.2007.09.007 17980556 10.1016/j.eplepsyres.2007.09.007

[13] Gagnon JF, Fantini ML, Bedard MA, Petit D, Carrier J, Rompre S, Decary A, Panisset M, Montplaisir J (2004) Association between waking EEG slowing and REM sleep behavior disorder in PD without dementia. Neurology 62(3):401–406. 10.1212/01.wnl.0000106460.34682.e9 14872020 10.1212/01.wnl.0000106460.34682.e9

[14] Tinuper P, Bisulli F, Cross JH, Hesdorffer D, Kahane P, Nobili L et al (2016) Definition and diagnostic criteria of sleep-related hypermotor epilepsy. Neurology 86(19):1834–1842 27164717 10.1212/WNL.0000000000002666 PMC4862248

[15] Kwan P, Arzimanoglou A, Berg AT, Brodie MJ, Allen Hauser W, Mathern G, Moshé SL, Perucca E, Wiebe S, French J (2010) Definition of drug resistant epilepsy: consensus proposal by the ad hoc task force of the ILAE commission on therapeutic strategies. Epilepsia 51(6):1069–1077. 10.1111/j.1528-1167.2009.02397.x 19889013 10.1111/j.1528-1167.2009.02397.x

[16] Cesari M, Heidbreder A, Gaig C, Bergmann M, Brandauer E, Iranzo A, Holzknecht E, Santamaria J, Högl B, Stefani A (2023) Automatic analysis of muscular activity in the flexor digitorum superficialis muscles: a fast screening method for rapid eye movement sleep without Atonia. Sleep 46(3):zsab299. 10.1093/sleep/zsab299 34984464 10.1093/sleep/zsab299 PMC9995778

[17] Troester MM, Quan SF, Berry RB, for the American Academy of Sleep Medicine et al (2023) The AASM manual for the scoring of sleep and associated events: rules, terminology and technical specifications. Version 3. American Academy of Sleep Medicine, Darien, IL

[18] Peter-Derex L, Klimes P, Latreille V, Bouhadoun S, Dubeau F, Frauscher B (2020) Sleep disruption in epilepsy: ictal and interictal epileptic activity matter. Ann Neurol 88(5):907–920. 10.1002/ana.25884 32833279 10.1002/ana.25884

[19] Nuwer MR, Corni G, Emerson R, Fuglsang-Frederiksen A, Guérit JM, Hinrichs H, Ikeda A, Luccas FJ, Rappelsburger P (1998) IFCN standards for digital recording of clinical EEG. Recommendations for the practice of clinical neurophysiology: guidelines of the international federation of clinical physiology. Electroenceph Clin Neurophysiol 106(3):259–261. 10.1016/s0013-4694(97)00106-5 9743285 10.1016/s0013-4694(97)00106-5

[20] Khalsa SBS, Conroy DA, Duffy JF, Czeisler CA, Dijk D-J (2002) Sleep-and circadian dependent modulation of REM density. J Sleep Res 11(1):53–59. 10.1046/j.1365-2869.2002.00276.x 11869427 10.1046/j.1365-2869.2002.00276.x

[21] Fantini ML, Michaud M, Gosselin N, Lavigne G, Montplaisir J (2002) Periodic leg movements in REM sleep behavior disorder and related autonomic and EEG activation. Neurology 59(12):1889–1894. 10.1212/01.wnl.0000038348.94399.f6 12499479 10.1212/01.wnl.0000038348.94399.f6

[22] Jo H, Kim D, Song J, Choi S, Joo E (2021) Sleep disturbances and phenoconversion in patients with REM sleep behavior disorder. J Clin Med 10(20):4709. 10.3390/jcm10204709 34682832 10.3390/jcm10204709 PMC8536960

[23] GBD 2016 Epilepsy Collaborators (2019) Global, regional, and National burden of epilepsy, 1990–2016: a systematic analysis for the global burden of disease study 2016. Lancet Neurol 18(5):e4. 10.1016/S1474-4422(19)30120-30126

[24] Lee K, Baron K, Soca R, Attarian H (2016) The prevalence and characteristics of REM sleep without Atonia (RSWA) in patients taking antidepressants. J Clin Sleep Med 12(3):351–355. 10.5664/jcsm.5582 26446247 10.5664/jcsm.5582 PMC4773631

[25] Schiller K, von Ellenrieder N, Avigdor T, El Kosseifi C, Abdallah C, Minato E, Gotman J, Frauscher B (2023) Focal epilepsy impacts rapid eye movement sleep microstructure. Sleep 46(2):zsac250. 10.1093/sleep/zsac250 36242588 10.1093/sleep/zsac250 PMC9905780

[26] Yeh WC, Li YS, Hsu CY (2023) Reduction in the propensity of rapid eye movement sleep and phasic-to-tonic ratio in patients with refractory epilepsy. Sleep 46(7):zsad115. 10.1093/sleep/zsad115 37075811 10.1093/sleep/zsad115

[27] Usami K, Matsumoto R, Kobayashi K, Hitomi T, Matsuhashi M, Shimotake A, Kikuchi T, Yoshida K, Kunieda T, Mikuni N, Miyamoto S, Takahashi R, Ikeda A (2017) Phasic REM transiently approaches wakefulness in the human Cortex-A Single-Pulse electrical stimulation study. Sleep 40(8). 10.1093/sleep/zsx077 10.1093/sleep/zsx077 28482107

[28] Simor P, Szalárdy O, Gombos F, Ujma PP, Jordán Z, Halász L, Erőss L, Fabó D, Bódizs R (2021) REM sleep microstates in the human anterior thalamus. J Neurosci 41(26):5677–5686. 10.1523/JNEUROSCI.1899-20.2021 33863786 10.1523/JNEUROSCI.1899-20.2021 PMC8244978

[29] Luppi PH, Clément O, Sapin E, Gervasoni D, Peyron C, Léger L, Salvert D, Fort P (2011) The neuronal network responsible for Paradoxical sleep and its dysfunctions causing narcolepsy and rapid eye movement (REM) behavior disorder. Sleep Med Rev 15:153–163. 10.1016/j.smrv.2010.08.002 21115377 10.1016/j.smrv.2010.08.002

[30] Antelmi E, Pizza F, Franceschini C, Ferri R, Plazzi G (2020) REM sleep behavior disorder in narcolepsy: A secondary form or an intrinsic feature? Sleep Med Rev 50:101254. 10.1016/j.smrv.2019 31931470 10.1016/j.smrv.2019.101254

[31] Grenot M, Roman A, Villalba M, Morel AL, Fort P, Arthaud S, Libourel PA, Peyron C (2024) Major alteration of motor control during rapid eye movements sleep in mice models of sleep disorders. Sleep 47(11):zsae178. 10.1093/sleep/zsae178 39121093 10.1093/sleep/zsae178

[32] Vanini G (2024) Are orexins-hypocretins the culprit for rapid eye movement sleep behavior disorder in narcolepsy? Sleep 47(11):zsae208. 10.1093/sleep/zsae208 39238164 10.1093/sleep/zsae208 PMC11543618

[33] Li HT, Viskaitis P, Bracey E, Peleg-Raibstein D, Burdakov D (2024) Transient targeting of hypothalamic orexin neurons alleviates seizures in a mouse model of epilepsy. Nat Commun 15(1):1249. 10.1038/s41467-024-45515-5 38341419 10.1038/s41467-024-45515-5 PMC10858876

